# Prevalence, type, and correlates of trauma exposure among adolescent men and women in Soweto, South Africa: implications for HIV prevention

**DOI:** 10.1186/s12889-016-3832-0

**Published:** 2016-11-25

**Authors:** Kalysha Closson, Janan Janine Dietrich, Busi Nkala, Addy Musuku, Zishan Cui, Jason Chia, Glenda Gray, Nathan J. Lachowsky, Robert S. Hogg, Cari L. Miller, Angela Kaida

**Affiliations:** 1 Faculty of Health Sciences, Simon Fraser University (SFU), Blusson Hall Rm 10522, 8888 University Drive, Burnaby, BC V5A 1S6 Canada; 2 British Columbia Centre for Excellence in HIV/AIDS, Vancouver, Canada; 3 Perinatal HIV Research Unit (PHRU), Faculty of Health Sciences, University of the Witwatersrand, Johannesburg, South Africa; 4 Faculty of Humanities, University of the Witwatersrand, Johannesburg, South Africa; 5 School of Public Health and Social Policy, University of Victoria, Victoria, Canada

**Keywords:** Adolescent, Young adult, Youth, HIV, Prevention, Trauma, Potentially traumatic events, Sexual and reproductive health, South Africa

## Abstract

**Background:**

Youth trauma exposure is associated with syndemic HIV risk. We measured lifetime prevalence, type, and correlates of trauma experience by gender among adolescents living in the HIV hyper-endemic setting of Soweto, South Africa.

**Methods:**

Using data from the Botsha Bophelo Adolescent Health Survey (BBAHS), prevalence of “ever” experiencing a traumatic event among adolescents (aged 14–19) was assessed using a modified Traumatic Event Screening Inventory-Child (TESI-C) scale (19 items, study alpha = 0.63). We assessed self-reported number of potentially traumatic events (PTEs) experienced overall and by gender. Gender-stratified multivariable logistic regression models assessed independent correlates of ‘high PTE score’ (≥7 PTEs).

**Results:**

Overall, 767/830 (92%) participants were included (58% adolescent women). Nearly all (99.7%) reported experiencing at least one PTE. Median PTE was 7 [Q1,Q3: 5-9], with no gender differences (*p* = 0.19). Adolescent men reported more violent PTEs (e.g., “seen an act of violence in the community”) whereas women reported more non-violent HIV/AIDS-related PTEs (e.g., “family member or someone close died of HIV/AIDS”). High PTE score was independently associated with high food insecurity among adolescent men and women (aOR = 2.63, 95%CI = 1.36-5.09; aOR = 2.57, 95%CI = 1.55-4.26, respectively). For men, high PTE score was also associated with older age (aOR = 1.40/year, 95%CI = 1.21-1.63); and recently moving to Soweto (aOR = 2.78, 95%CI = 1.14-6.76). Among women, high PTE score was associated with depression using the CES-D scale (aOR = 2.00, 95%CI = 1.31-3.03,) and inconsistent condom use *vs*. no sexual experience (aOR = 2.69, 95%CI = 1.66-4.37).

**Conclusion:**

Nearly all adolescents in this study experienced trauma, with gendered differences in PTE types and correlates, but not prevalence. Exposure to PTEs were distributed along social and gendered axes. Among adolescent women, associations with depression and inconsistent condom use suggest pathways for HIV risk. HIV prevention interventions targeting adolescents must address the syndemics of trauma and HIV through the scale-up of gender-transformative, youth-centred, trauma-informed integrated HIV and mental health services.

**Electronic supplementary material:**

The online version of this article (doi:10.1186/s12889-016-3832-0) contains supplementary material, which is available to authorized users.

## Background

South Africa has one of the highest rates of HIV globally, with an adult prevalence of 17.9% [1]. HIV disproportionately affects young people, and young women in particular. Among youth aged 15 to 24 years of age, 13.3% of young women and 3.8% of young men are living with HIV [2]. Addressing the high rate and burden of HIV among South African youth, and adolescent women in particular [3], is a national and global public health priority. While efforts are underway to scale-up access to several biomedical HIV prevention tools, including pre-exposure prophylaxis (PrEP), antiretroviral therapy (ART) for prevention (‘TasP’), medical male circumcision, and female and male condoms [3, 4], demand for these programs will be shaped by the broader developmental, social and structural forces which influence adolescent sexual behaviour [5]. At present, there is a lack of literature on gendered differences in prevalence, types and influence of traumatic experiences and their relationship with adolescent HIV risk.

Experiences of childhood trauma are common among adolescents in South Africa, with estimates of physical and sexual violence in childhood ranging from 1.6–54.2% [6]. Traumatic experiences in childhood and adolescence have serious implications for short and long-term psychological and physical health outcomes, and have been associated with increased incidence of HIV [7–11]. The pathway from trauma and depression to heightened risk of HIV and other sexually transmitted infections has been described through the negative effects of depression on impulse control, risk perception [12], self-esteem and self-efficacy [13], substance use [14], and socio-structural vulnerability [15], which compromise HIV prevention behaviours [16, 17]. Such pathways are highly gendered, with both the prevalence of depression and associations with increased risk of condomless sex shown to be higher among adolescent women than adolescent men [18].

The disproportionate exposure to potentially traumatic events (PTEs) experienced by people living with HIV (PLHIV), has been referred to as a syndemic (“synergistically interacting epidemics”) [19], yielding a range of poor social, clinical, and public health outcomes, including decreased social functioning, elevated rates of post-traumatic stress disorder (PTSD), increased prevalence of high-risk sexual and drug use behaviours, suboptimal adherence to ART, poor HIV clinical outcomes, increased HIV transmission risk, and higher mortality [7, 9, 10]. Little attention, however, has focused on gendered impacts and the presence of syndemic risks which can have a multiplicative effect on HIV risk [20], including multiple types of PTEs (e.g. physical, sexual, and emotional) [21].

Adolescent men and women are exposed to different types and consequences of trauma, particularly with respect to violent and non-violent forms. Globally, violence against women is a major social justice issue [22, 23], an under-addressed public health priority, and an established risk factor for HIV acquisition and other negative health outcomes [3, 24]. In South Africa, where reports of violence are known to under-estimate the true prevalence [25], 20% of women attending antenatal care reported experiencing sexual violence, among the highest prevalence in the world [22, 26]. Among adolescent men, experiences of perpetrating or witnessing interpersonal violence drive rates of trauma exposure [11, 24, 27]. This is significant as earlier research among South African adolescent men demonstrated an association between witnessing community violence and high sexual HIV risk behaviours such as multiple concurrent sexual partnerships [28].

The effects of experiencing trauma on mental health and coping strategies also differ between adolescent men and women in ways that influence HIV risk pathways. For instance, PTEs experienced by South African women have been shown to increase internalized behaviours such as depression, anxiety and PTSD [23, 29], which synergistically contribute to increased risk for HIV and other sexually transmitted infections (STIs) [26, 30]. However, adolescent men are more likely to respond to PTEs with adverse externalized behaviours that introduce HIV risk, including delinquency, aggression and substance abuse [21]. This distinction in type of PTEs and behavioural responses demands gender-specific analysis, support, and response.

We measured the lifetime prevalence and correlates of PTEs overall, and by gender among adolescent men and women in Soweto, South Africa. This information is critical to inform youth-centred sexual and reproductive health and HIV prevention programming that considers the broader risk environments that youth navigate [31].

## Methods

### Study setting

We used cross-sectional survey data from adolescents (aged 14–19 years) enrolled in the Botsha Bophelo Adolescent Health Study (BBAHS) in Soweto, South Africa. Soweto is a large township southwest of Johannesburg with a population of approximately 1.3 million predominantly (98.5%) black inhabitants residing in informal and formal settlements [32]. While there are no population-level statistics on HIV prevalence among adolescents in Soweto, a recent study of 11,552 adolescents and young adults (14–25 years) residing in Soweto, reported that 4% of those who accessed HIV testing services at a local youth-centered clinic tested positive for HIV, including 2% of young men 4% of young women [33].

BBAHS was conducted at the Perinatal Health Research Unit (PHRU) and the Kganya Motsha Adolescent Centre (KMAC) in Soweto, South Africa. KMAC was opened in 2008 with a local mandate to address HIV and sexual and reproductive health priorities of adolescents (ages 14–19 years). Earlier pilot studies on adolescent health identified the urgent need for such youth-centred services, and informed the development and implementation of BBAHS [33–36].

### Study participants

Adolescents aged 14–19 years residing in Soweto were eligible to participate in BBAHS. Participants were recruited from across 41 townships to be representative of adolescents living in formal and informal communities within Soweto. Participant recruitment occurred around local malls, schools, neighbourhood hangouts, through peer-word-of-mouth, and staff outreach. We used a targeted stratified sampling and recruitment approach, based on geographic location, age, and gender. In order to reflect the gendered dimensions of HIV risk in South Africa, we aimed for a sample comprised of 60% young women and 40% young men. The research team approached interested adolescents for participation, and if eligible, were enrolled in the study. A total of 956 interviews were completed between March 2010 and March 2012. This amount of recruitment time was required to meet stratified sampling targets, and to ensure inclusion of youth from more remotely located townships with Soweto and harder-to-reach youth sub-populations. Of 956 completed interviews, n = 126 were excluded as they were determined to be outside of the targeted age criteria or had incomplete data, yielding a final sample of 830 adolescent participants. Additional information about the study procedures of the BBAHS can be found elsewhere [37].

### Ethical considerations

Adolescents under 18 years signed an informed assent form and provided a signed informed consent form from a parent or legal guardian. Adolescents aged 18 or 19 signed an informed consent form. Age was verified using birth certificates or other identity documents.

Ethical approval for the study was granted by the ethics committees of the University of the Witwatersrand (Johannesburg, South Africa) and Simon Fraser University (Burnaby, Canada).

### Data collection

An interviewer-administered, structured, online questionnaire was delivered to participants (supported by SurveyMonkey^TM^ software) via iPad or desktop computer. Interviewers received extensive training in good clinical practice guidelines, participant recruitment, administering questionnaires, and participant referral in cases where additional support was required after the study visit. Interviews were conducted in either English or isiZulu at the PHRU, the KMAC, or at a private location selected by the participant. Questionnaires took an average of 60 min to complete, and participants received 50 Rand (approximately 7 USD at the time) as compensation for their time and transportation costs. An international team of experts in adolescent health and HIV, including an adolescent Community Advisory Board (CAB), contributed to the development of the BBAHS questionnaire [37].

### Measures

#### Primary outcome: trauma experience

Assessment of ‘trauma experience’ followed Norris’ [29] comprehensive definition of traumatic events as “*any event that produces symptoms of traumatic stress*” (23, p. 409). We measured PTEs using a modified version of the Traumatic Events Screening Inventory–Child (TESI-C) [29]. Unlike other trauma scales, the TESI-C scale was developed to be language appropriate for children and youth.

The TESI-C measures the history of trauma by asking about exposure (“yes” *vs.* “no”) to twenty PTEs including “injuries, hospitalizations, domestic violence, community violence, disasters, accidents, physical abuse and sexual abuse” [38]. Historically, this scale has been used in child and adolescent psychological screening [38]. For our study, the TESI-C items were modified to account for the social context and physical environment of adolescents in Soweto [38]. For example, TESI-C items regarding natural disasters, acts of war or terrorism, kidnapping and animal attacks were omitted. Similar to other South African studies examining the impact of traumatic experiences in adolescents, we added items regarding parents separating, parents arguing, changing schools, parents’ job security, family members with HIV/AIDS, family members dying of HIV/AIDS, discrimination, financial security, personal physical attack were added. The final adapted scale included a total of 19 items (study alpha = 0.63; Table 2). A comparison of items from the original TESI-C scale and the modified version used in this analysis is included in the Additional file 1.

We measured prevalence of experiencing a potentially traumatic event (i.e., a response of “Yes” to one or more of the 19 items included in the modified TESI-C scale) overall and by gender. We also assessed number of reported PTEs and calculated a PTE score (range = 0-19), with higher scores indicating higher PTE experience. Scores greater than the scale median were considered ‘high PTE score’ vs. ‘low PTE score’.

### Explanatory factors

#### Socio-demographic characteristics

We assessed socio-demographic characteristics by gender (adolescent man vs. adolescent woman), age in years (continuous), ethnicity (Zulu, Xhosa, Sotho, Tswana or other), education (high school or greater vs. less than high school), and employment (student vs. unemployed vs. employed [full-time/part-time/self-employed]). Additional determinants of socio-economic status included length of time living in Soweto (<5 years vs. ≥5 years vs. since birth), housing type (brick house or flat owned by family vs. brick house or flat rented by family or other housing type vs. reconstructive development housing [RDP] or shack), food insecurity (low vs. medium vs. high, measured via a 9-item hunger and food security scale [39] [study Cronbach’s α = 0.81]), and receiving a household social grant in the past 12-months (yes vs. no; including disability, age pension, child support or other social grant), and history of incarceration (ever vs. never).

#### Depression

The 20-point Center for Epidemiologic Studies Depression (*CES-D)* Scale was utilized to measure probable depression (study Cronbach’s α = 0.81, range = 0-60, with higher scores indicating greater depressive symptoms) [40]. In the general population the American Psychological Association suggests using a cut off of 16 or higher to determine major depressive disorder [41]. We chose a higher cut off of ≥24 as this has been previously described as the best cut-off to determined ‘probable depression’ among adolescents [18, 42].

#### Sexual behaviour

History of sexual activity was defined by participant report of ever having had intercourse (yes vs. no), current sexual activity was defined as having had sex (vaginal or anal) in the 6 months prior to interview (yes vs. no) and, if yes, whether the participant had more than one sexual partner in the last 6 months (yes vs. no). Consistent condom use was assessed via self-reported lifetime use during anal and/or vaginal sex, as applicable, and frequency (always vs.vs sometimes vs. never) in the 6 months prior to interview (lifetime consistent condom use vs. any inconsistent or no condom use vs. never had sex). History of STI diagnosis and/or symptoms (ever vs. never), history of HIV testing (ever vs. never), and HIV status (HIV-positive vs. HIV-negative vs. unknown HIV status) was assessed via self-report.

#### Substance use

We assessed self-reported frequency of alcohol use in the 6 months prior to interview (once a month or more vs. less than once a month or never). We also assessed any use of illicit (e.g., heroin, cocaine, ecstasy) or licit drugs used in a manner other than which they are prescribed (e.g., prescription pills, antiretrovials/whoonga), excluding marijuana in the 6 months prior to interview (yes vs. no). Use of marijuana (yes vs. no) was assessed separately, given different patterns of use among youth [43, 44].

### Statistical analysis

All analyses were conducted using SAS 9.4, stratified by self-identified gender. Descriptive statistics (median, 1^st^ quartile [Q1] and 3^rd^ quartile [Q3] for continuous variables and n, % for categorical variables) were used to characterize baseline distributions of study variables. Differences in baseline variables and trauma scores by gender were compared using Wilcoxon rank sum test for continuous variables and Pearson *χ* ^2^ or Fisher’s exact test for categorical variables.

Univariable and multivariable logistic regression were used to identify variables associated with high PTE score, separately for adolescent men and women. Variables of interest with univariable *p*-values <0.20 were included in multivariable model selections. After testing for collinearity, only the sexual behaviour variable ‘inconsistent condom use (yes vs. no vs. never had sex)’ was considered for inclusion in the final model. For all other variables, model selections were performed using backward selection based on Type III *p*-values to reach the optimal (minimized) AIC. All statistical tests were considered statistically significant at α < 0.05.

## Results

### Baseline characteristics

Of 830 participants, 767 answered all 19 TESI-C items and were included in this analysis of whom 442 (58%) were adolescent women and 325 (42%) were adolescent men (Table 1). Median age was 17 years [Q1-Q3: 16-18], 45% were Zulu, 85% were currently enrolled in school, and 6% had ever been incarcerated. A majority had lived in Soweto since birth (77%), lived in brick house/flat owned by the family (71%), reported high food insecurity (52%), and lived in a household which had received a social grant in the last 12 months (57%).

**Table 1:** Baseline characteristics of participants (aged 14–19 years) overall and by gender (*n* = 767)

Baseline characteristics	Overall (*n* = 767)		Adolescent Men (*n* = 325)		Adolescent Women (*n* = 442)		*p*-value
	*n*	%	*n*	%	*n*	%	
Socio-demographic characteristics							
*Age at interview (years, median, Q1,Q3)*	*17*	*16,18*	*17*	*16,18*	*18*	*16,18*	*0.197*
Years lived in Soweto							
< 5 years	71	9.4	27	8.4	44	10.0	0.347
≥ 5 years	106	14.0	51	15.9	55	12.5	
Since birth	582	76.7	242	75.6	340	77.5	
missing	8		5		3		
Ethnicity							
Zulu	345	45.0	166	51.1	179	40.5	**0.005**
Xhosa	92	12.0	39	12.0	53	12.0	
Sotho	124	16.2	40	12.3	84	19.0	
Tswana	85	11.1	26	8.0	59	13.4	
Other ethnicities	121	15.8	54	16.6	67	15.2	
Education							
≥ High school	9	1.2	7	2.2	2	0.5	**0.041**
< High school	758	98.8	318	97.9	440	99.6	
Employment							
Student	649	85.1	264	81.5	385	87.7	0.056
Unemployed	85	11.1	44	13.6	41	9.3	
Employed	29	3.8	16	4.9	13	3.0	
Missing	<5		<5		<5		
Housing							
Brick house/Flat owned by family	547	71.3	220	67.7	327	74.0	0.160
Brick house/Flat rented by family/other	18	2.3	9	2.8	9	2.0	
RDP house/Shack	202	26.3	96	29.5	106	24.0	
Food Insecurity							
Low	169	22.0	59	18.2	110	24.9	0.078
Medium	203	26.5	88	27.1	115	26.0	
High	395	51.5	178	54.8	217	49.1	
Household Social Grant in the last 12 months							
No	325	42.9	141	44.3	184	41.9	0.506
Yes	432	57.1	177	55.7	255	58.1	
missing	10		7		3		
Incarceration history							
No	646	93.8	258	91.2	388	95.6	**0.019**
Yes	43	6.2	25	8.8	18	4.4	
Missing	78		42		36		
Sexual behaviour and HIV variables							
Ever had sex							
No	338	44.1	116	35.7	222	50.2	**<.001**
Yes	429	55.9	209	64.3	220	49.8	
Sexually Active in the past 6 months (L6M)^a^							
No	153	36.5	80	39.6	73	33.6	0.205
Yes	266	63.1	122	60.4	144	66.4	
missing	10		7		3		
Number of partners (among those reporting sexual activity in L6M)^b^							
1 partner	168	64.6	51	43.6	117	81.8	**<.001**
≥ 2 partner	92	35.4	66	56.4	26	18.2	
Missing	6						
Condom use^a^							
Consistent condom use	189	46.3	93	47.2	96	45.5	0.729
Inconsistent condom use	219	53.7	104	52.8	115	54.5	
missing	21		12		9		
HIV testing history							
No	414	54.1	187	57.7	227	51.5	0.087
Yes	351	45.9	137	42.3	214	48.5	
HIV status (self-report)							
HIV-positive	11	1.4	5	1.5	6	1.4	0.187
HIV-negative	329	42.9	127	39.1	202	45.7	
Unknown/never tested	427	55.7	193	59.4	234	52.9	
STI or STI symptomology^a^							
No	332	77.4	173	82.8	159	72.3	**<.001**
Yes	97	22.6	36	17.2	61	27.7	
Substance use and mental health variables							
Alcohol use in the last 6 months (L6M)							
No	267	34.99	104	32.1	163	37.1	0.150
Yes	496	65.01	220	67.9	276	62.9	
Drug use in L6M (excluding marijuana use)							
No	728	94.9	297	91.4	431	97.5	**<.001**
Yes	39	5.1	28	8.6	11	2.5	
Probable Depression							
No	510	66.5	229	70.5	281	63.6	**0.046**
Yes (CES-D score ≥ 24)	257	33.5	96	29.5	161	36.4	

Note: *p*-values in bold are significant (<.05)

*Abbreviations: CES-D* center for epidemiologic studies- depression scale, *RDP* reconstruction and development programme, *STI* sexually transmitted infection, *HIV* human immunodeficiency virus

^a^Among those reporting sexual activity ever

^b^Among those reporting sexual activity in the last 6 month

Overall, 56% of participants reported having ever had sex, including 64% of adolescent men and 50% of adolescent women (*p* < 0.001 for gender difference). Of those reporting sexual activity in the six months prior to the interview, 35% reported having more than one sexual partner in the previous 6 months (including 56% of adolescent men and 18% of adolescent women [*p* < 0.001]). Among those who had ever had sex, 54% reported inconsistent condom use (including 53% of adolescent men and 55% of adolescent women [*p* = 0.729]) and 23% reported ever having been diagnosed with an STI or experienced STI symptoms (including 17% of adolescent men and 28% of adolescent women [*p* = 0.009]). Overall, 1.4% reported being HIV-positive (1.5% of adolescent men and 1.4% of women, *p* = 0.19).

In the six months prior to interview, nearly two-thirds (65%) reported alcohol use and 5% reported using other drugs. One-third (34%) had probable depression, with higher rates among adolescent women than men (36% vs. 30%, *p* = 0.05).

### Experience of potentially traumatic events (PTEs)

Nearly all participants (99.7%) reported experiencing at least 1 PTE. Median number of PTEs experienced was 7 [Q1-Q3: 5-9], with no significant difference by gender (*p* = 0.19). Overall, 47% of adolescent men and 45% of adolescent women experienced a high PTE score (≥7 events (*p* = 0.603)).

Table 2 shows the proportion of adolescents who reported experiencing each of the 19 PTE items included in the adapted TESI-C scale by gender. Nearly three-quarters (74%) of adolescent men and women reported experiencing the death of a family member or someone close to them. Over two-thirds (68%) had witnessed a close family member or friend deal with a serious illness or injury. Nearly half reported that their parents were separated or divorced (48%) or that their family struggled with money (46%). In general, adolescent men were more likely to have experienced or perpetuated violent forms of traumatic experiences (e.g. forcing someone to have sex with them [7%], deliberately inflicting harm on another [51%], witnessed an act of violence in the community [76%]). Adolescent women were more likely to experience psychological and emotional experiences of potentially traumatic events (e.g. having a family member have [46%] or die from [41%] HIV/AIDS).

**Table 2:** Prevalence of potentially trauma event (PTE) experiences among participants (14–19 years) overall and by gender (*n* = 767)

	Overall (*n* = 767)		Adolescent Men (*n* = 325)		Adolescent Women (*n* = 442)		*p*-value
	*n*	%	*n*	%	*n*	%	
Experienced at least one PTE	765	99.7	325	100.0	440	99.6	0.511
High trauma score (≥7) (alpha = 0.63)	348	45.4	151	46.5	197	44.6	0.603
Separated from mom (e.g. lived with another relative or in foster care)	253	33.0	118	36.3	135	30.5	0.093
Parents separated	370	48.2	153	47.1	217	49.1	0.581
Parents argued frequently or more than usual	259	33.8	111	34.2	148	33.5	0.846
Changed schools (not because of graduation) or moved to a new home	245	31.9	123	37.9	122	27.6	**0.003**
Parent/guardian lost job	342	44.6	139	42.8	203	45.9	0.385
Lost home or had no home	65	8.5	38	11.7	27	6.1	**0.006**
Family member or someone close had HIV/AIDS	287	37.4	85	26.2	202	45.7	**<0.001**
Family member or someone close died of HIV/AIDS	273	35.6	91	28.0	182	41.2	**0.001**
Family member or someone close died	569	74.2	243	74.8	326	73.8	0.751
Family member or someone close was very sick or had a bad injury	524	68.3	230	70.8	294	66.5	0.211
Experienced race/ethnicity discrimination	183	23.9	77	23.7	106	24.0	0.926
Family struggled with money	355	46.3	147	45.2	208	47.1	0.616
Seen an act of violence towards someone else (not in family)	538	70.1	248	76.3	290	65.6	**0.001**
Experienced an act of violence by someone not in your family	316	41.2	147	45.2	169	38.2	0.052
Seen an act of violence in the family	324	42.2	136	41.9	188	42.5	0.849
Experienced an act of violence by someone in your family	240	31.3	107	32.9	133	30.1	0.403
Deliberately inflicted harm on another person	293	38.2	166	51.1	127	28.7	**<0.001**
Experienced forced Sex	98	12.8	35	10.8	63	14.3	0.153
Experienced forcing someone to have sex	30	3.9	24	7.4	6	1.4	**<0.001**

Note: *p*-values in bold are significant (>.05)

Overall, 14% of adolescent women and 11% of adolescent men reported experiencing forced sex (*p* = 0.153) while 1.4% and 7.4% reported ever forcing someone to have sex with them (*p* < 0.001).

### Correlates of high PTE scores

In unadjusted models among adolescent men (see Table 3), high PTE score was associated with older age, living in Soweto for <5 years, self-reported Tswana ethnicity, high food insecurity, drug use in the past six months, sexual experience, and inconsistent condom use. In the adjusted model (see Table 3), adolescent men with high PTE scores had significantly higher adjusted odds of being older (aOR = 1.40/year, 95%CI = 1.21-1.63); recently moving to Soweto (<5 years) vs. living in Soweto ‘since birth’ (aOR = 2.78, 95%CI = 1.14-6.76); and high vs. low food insecurity (aOR = 2.63 95%CI = 1.36-5.09).

**Table 3:** Univariate and adjusted analysis of variables associated with high PTE scores among adolescent men (*n* = 325)

	Low PTE score		High PTE score		*p*-value	High PTE score vs. Low PTE score					
Variables	*n*	%	*n*	%	Wilcoxon/Chisq	OR	95% CI		AOR	95% CI	
Socio-demographic characteristic											
*Age at interview (per year, median Q1,Q3)*	*17*	*15,18*	*18*	*16,18*	***<.001***	*1.37*	*1.19*	*1.59*	***1.40***	***1.21***	***1.63***
Years lived in Soweto											
Since birth	133	76.9	109	74.2	0.059	Ref			Ref		
≥ 5 years	31	17.9	20	13.6		0.79	0.42	1.46	0.75	0.39	1.43
< 5 years	9	5.2	18	12.2		2.44	1.05	5.65	**2.78**	**1.14**	**6.76**
Ethnicity											
Zulu	99	56.9	67	44.4	0.174	Ref					
Xhosa	18	10.3	21	13.9		1.72	0.85	3.48	Not Selected		
Sotho	21	12.1	19	12.6		1.34	0.67	2.67			
Tswana	10	5.8	16	10.6		2.36	1.01	5.52			
Other ethnicities	26	14.9	28	18.5		1.59	0.86	2.95			
Employment											
Student	147	85.0	117	77.5	0.193	Ref					
Unemployed	20	11.56	24	15.89		1.51	0.79	2.86	Not Selected		
Employed	6	3.5	10	6.6		2.09	0.74	5.93			
Housing											
Brick house/Flat owned by family	123	70.7	97	64.2	0.414	Ref					
Brick house/Flat rented by family/Hostel/Other	5	2.9	4	2.7		1.01	0.27	3.88			
RDP house/Shack	46	26.44	50	33.11		1.38	0.85	2.23			
Food Insecurity											
Low	39	22.4	20	13.3	**0.026**	Ref			Ref		
Medium	51	29.3	37	24.5		1.41	0.71	2.81	1.58	0.76	3.29
High	84	48.3	94	62.3		2.18	1.18	4.03	**2.63**	**1.36**	**5.09**
Household Social Grant											
No	81	47.9	60	40.3	0.170	Ref			Not Selected		
Yes	88	52.1	89	59.7		1.37	0.87	2.13			
Incarceration history											
No	148	92.5	110	89.4	0.367	Ref					
Yes	12	7.5	13	10.6		1.46	0.64	3.32			
Sexual behaviour and HIV											
HIV testing history											
No	99	57.2	88	58.3	0.848	Ref					
Yes	74	42.8	63	41.7		0.96	0.62	1.49			
HIV Result											
Positive	3	1.7	2	1.3	0.940	Ref					
Negative	69	39.7	58	38.4		1.26	0.20	7.81			
Unknown/Never tested	102	58.6	91	60.3		1.34	0.22	8.19			
Sex Ever											
No	77	44.3	39	25.8	**0.001**	Ref			Not included^a^		
Yes	97	55.8	112	74.2		2.28	1.42	3.65			
Ever STI											
No	85	48.9	88	58.3	**0.001**	Ref			Not included^a^		
Yes	12	6.9	24	15.9		1.93	0.91	4.11			
Never had sex	77	44.3	39	25.8		0.49	0.30	0.80			
Sexually Active P6M											
No	41	24.1	39	26.4	**0.001**	Ref			Not Included^a^		
Yes	52	30.6	70	47.3		1.42	0.80	2.49			
Never had sex	77	45.3	39	26.4		0.53	0.30	0.95			
Inconsistent condom use											
Never had sex	77	45.8	39	26.9	**0.002**	Ref			Not Selected		
No	44	26.2	49	33.8		2.20	1.26	3.85			
Yes	47	28.0	57	39.3		2.39	1.39	4.13			
More than 1 partner in the L6M											
No	23	13.6	28	19.4	**0.016**	Ref			Not Included^a^		
Yes	28	16.6	38	26.4		1.11	0.53	2.33			
Never had sex/Sexually inactive	118	69.8	78	54.2		0.54	0.29	1.01			
Substance use and mental health variables											
Alcohol use in L6M											
No	63	36.4	41	27.2	0.075	1.09	0.47	2.52			
Yes	110	63.6	110	72.9		0.65	0.28	1.51			
Probable Depression											
No	129	74.1	100	66.2	0.119	Ref			Not Selected		
Yes (score ≥ 24)	45	25.9	51	33.8		1.54	0.96	2.47			
Drug use ever in L6M (excluding marijuana use)											
No	165	94.8	132	87.4	**0.018**	Ref			Not Selected		
Yes	9	5.2	19	12.6		2.64	1.16	6.02			

Note: AORs and *p*-values in bold are significant (<.05)

*Abbreviations: CI* confidence intervals, *OR* odds ratio, *AOR* adjusted odds ratio, *CES-D* center for epidemiologic studies- depression scale, *RDP* reconstruction and development programme, STI, sexually transmitted infection, *HIV* human immunodeficiency virus

^a^Not included due to Collinearity

In the unadjusted models among adolescent women (see Table 4), high PTE score was associated with, high food insecurity, incarceration history, received a household social grant in the last year, probable depression, sexual experience and inconsistent condom use. In the adjusted model (see Table 4), adolescent women with high PTE scores had significantly higher adjusted odds of high food insecurity (aOR = 2.57, 95%CI = 1.55-4.26); probable depression (aOR = 2.00, 95%CI = 1.31-3.03); and inconsistent condom use vs. no sexual experience (aOR = 2.69, 95%CI = 1.66-4.37).

**Table 4:** Univariate and adjusted analysis of variables associated with high PTE scores among adolescent women (*n* = 442)

	Low PTE score		High PTE score		*p*-value	High PTE score vs. Low PTE score					
Variables	*n*	%	*n*	%	Wilcoxon/Chisq	OR	95% CI		AOR	95% CI	
Socio-demographic characteristics											
*Age*	*17*	*16,18*	*18*	*16,18*	*0.182*	*1.10*	*0.97*	*1.24*	*Not Selected*		
Years lived in Soweto											
< 5 years	22	9.0	22	11.3	0.511	Ref					
≥ 5 years	28	11.5	27	13.9		0.96	0.44	2.13			
Since birth	194	79.5	146	74.9		0.75	0.40	1.41			
Ethnicity											
Zulu	104	42.5	75	38.1	0.764	Ref					
Xhosa	29	11.8	24	12.2		1.15	0.62	2.13			
Sotho	48	19.6	36	18.3		1.04	0.62	1.76			
Tswana	29	11.8	30	15.2		1.43	0.79	2.59			
Other	35	14.3	32	16.2		1.27	0.72	2.23			
Employment											
Student	217	89.7	168	85.3	0.379	Ref					
Unemployed	19	7.85	22	11.17		1.50	0.78	2.85			
Employed	6	2.5	7	3.6		1.51	0.50	4.57			
Housing											
House owned by family	184	75.1	143	72.6	0.577	Ref					
House rented by family/Other	6	2.5	3	1.5		0.64	0.16	2.62			
RDP house/Shack	55	22.45	51	25.89		1.19	0.77	1.85			
Food Insecurity											
Low	77	31.4	33	16.8	**<.001**	Ref			Ref		
Medium	71	29.0	44	22.3		1.45	0.83	2.52	1.49	0.84	2.65
High	97	39.6	120	60.9		2.89	1.77	4.70	**2.57**	**1.55**	**4.26**
Household ever Received Social Grant											
No	112	46.1	72	36.7	0.048	Ref			Not Selected		
Yes	131	53.9	124	63.3		1.47	1.00	2.16			
Sexual behaviour and HIV variables											
HIV testing history											
No	139	57.0	88	44.7	**0.010**	Ref			Not Selected		
Yes	105	43.0	109	55.3		1.64	1.12	2.39			
HIV Result											
Positive	3	1.2	3	1.5	0.131	Ref					
Negative	102	41.6	100	50.8		0.98	0.19	4.97			
Unknown	140	57.1	94	47.7		0.67	0.13	3.40			
Sex Ever											
No	142	58.0	80	40.6	**<.001**	Ref			Not included*		
Yes	103	42.0	117	59.4		2.02	1.38	2.95			
STI or STI symptomology											
No	80	32.7	79	40.1	**<.001**	Ref					
Yes	23	9.4	38	19.3		1.67	0.91	3.06			
Never had sex	142	58.0	80	40.6		0.57	0.38	0.86			
Sexually Active L6M											
No	41	16.9	32	16.3	**<.001**	Ref					
Yes	60	24.7	84	42.9		1.79	1.02	3.17			
Never had sex	142	58.4	80	40.8		0.72	0.42	1.24			
Inconsistent condom use											
Never had sex	142	59.7	80	41.0	**<.001**	Ref			Ref		
No	52	21.9	44	22.6		1.50	0.92	2.44	1.59	0.96	2.63
Yes	44	18.5	71	36.4		2.86	1.80	4.56	**2.69**	**1.66**	**4.37**
More than 1 partner in L6M											
No	49	20.2	68	34.9	**<.001**	Ref			Not included*		
Yes	11	4.5	15	7.7		0.98	0.42	2.32			
Never had sex/Sexually inactive	183	75.3	112	57.4		0.44	0.29	0.68			
Substance use and mental health variables											
Alcohol Use in the L6M											
No	102	42.0	61	31.1	**0.019**	Ref			Not Selected		
Yes	141	58.0	135	68.9		1.60	1.08	2.38			
Probable Depression											
No	176	71.8	105	53.3	**<.001**	Ref			**Ref**		
Yes (score ≥ 24)	69	28.2	92	46.7		2.23	1.51	3.32	**2.00**	**1.31**	**3.03**
Incarceration history											
No	226	97.4	162	93.1	**0.037**	Ref			Not Selected		
Yes	6	2.6	12	6.9		2.79	1.03	7.59			
Drug use ever in L6M (excluding marijuana use)											
No	239	97.6	192	97.5	0.952	Ref					
Yes	6	2.5	5	2.5		1.04	0.31	3.45			

Note: AORs in bold are significant (<.05)

*Abbreviations: CI* confidence intervals; *OR* odds ratio, *AOR* adjusted odds ratio, *CES-D* center for epidemiologic studies- depression scale, *RDP* reconstruction and development programme, *STI* sexually transmitted infection, *HIV* human immunodeficiency virus

*Not included due to Collinearity

## Discussion

Similar to other South African and African studies [8, 45], we found that adolescents in our study experienced high levels of PTEs. Nearly all participants experienced at least one PTE (99.7%) and had experienced on average 7 PTEs at the time of their interview with no differences by gender. A study of U.S adolescents (aged 13–17) found that 61.8% had lifetime PTE experience [46], compared with 99.7% of adolescents within our study. Among both adolescent men and women, increased exposure to PTE was associated with high levels of food insecurity. This finding has implications for sexual and reproductive health (SRH) outcomes and overall well-being for South African adolescent men and women faced with syndemic risks including high levels of community-level violence and sexual victimization [21]. In addition, our findings suggest no difference in the prevalence of PTEs between adolescent men and women, rather differences in the types of traumatic occurrences. Despite no significant differences in PTE prevalence by gender, we pursued a gender stratified analysis to enable examination of differential correlates of experiencing multiple PTEs. These findings highlight a need for future research to explore the differential potential gendered impacts of PTEs experienced among adolescents.

Consistent with previous literature, we found that PTE exposure and the effects are distributed along social and gendered axes. For example, a number of studies globally have found that young women are more likely to experience sexual assault while men are more likely to experience physical assault [29, 31, 45].

### Adolescent women

Our results align with previous research indicating that co-occuring multiple PTEs experienced by women influence heightened depression symptomology [8], and compound syndemic risks of HIV transmission through increased HIV risk behaviour such as inconsistent condom use [10, 30]. The synergistic effect of multiple experiences of PTEs and increased HIV acquisition risk may be exacerbated among women living in vulnerable urban environments, such as Soweto, facing economic hardships and high levels of food insecurity [23, 30]. These compounding experiences of structural vulnerability influence economic dependence - placing women in inferior roles in their relationships - in turn increasing experiences of gender-based violence, inability to negotiate condom use, and ultimately HIV transmission risk [3, 23].

### Adolescent men

Our results indicate that high-PTE scores were more commonly found among older adolescent men who have recently moved to Soweto, and who face high levels of food insecurity. Experiences of trauma can accumulate over the lifecourse, [47], as such older age was a hypothesized finding for higher number of PTEs among men in our study. The exposure to multiple experiences of PTEs at a young age have been found to perpetuate aggressive behaviour and negative views towards women in adulthood [48, 49]. The development of negative views towards women may perpetuate harmful gender norms and inequitable power dynamics in relationships, which has shown to have significant implications for the HIV epidemic in South Africa [24, 50–52]. Furthermore, young men living in South Africa face extremely high rates of interpersonal violence. A study assessing hospital data on injuries within the Mthatha Hospital Complex in South Africa, found that the majority of injuries occurred among men, with 60% of all cases being for acts of interpersonal violence [27]. Despite extremely high levels of PTEs within men participating in our study, we found that this was not significantly associated with increased depression symptomology or inconsistent condom use. Previous research has explored the relationship between high levels of trauma and post-traumatic growth [53]. Resilience to HIV risk among adolescent men living in HIV hyper-endemic nations experiencing concurrent poverty and high-levels of PTEs should be further explored.

### Intervention implications

Reducing syndemic risks to traumatic experiences in both adolescent men and women is likely to have a positive impact on HIV transmission through multiple pathways. The scale-up of community and structural level interventions, as well as increased focus on trauma-informed models of care for adolescents in South Africa is critical for addressing the HIV epidemic [21, 54]. For adolescent women, intervention strategies aimed at increasing economic independence, reducing gender-based violence, reducing inequities in relationship power and control, and challenging gender norms, are critical to increase sustained and widespread uptake of HIV prevention options, including male and female condoms and, in more recent years, pre-exposure prophylaxis (PrEP), [48, 55–57]. Among adolescent women, high rates of sexual violence and inequities in relationship power [50, 58, 59] intersect to compromise opportunities to negotiate condom use [30, 60–62]. Given demonstrated links between trauma, poor mental health, and sexual behaviours, mediated through pathways of gender and power inequity, central to the efforts to reduce HIV incidence among adolescent women is a clear need to scale-up access to youth-centred, trauma-informed, and women-controlled HIV prevention strategies, inclusive of PrEP [4].

Trauma-focused cognitive behavioural therapy (TF-CBT) has been shown to be highly beneficial in reducing sexual health risk. Hien and colleagues [63] implemented a skill-based TF-CBT program focusing on various domains including: personal self-management, coping, communication, boundary setting, HIV risk reduction and reducing unsafe behaviour in general. Women in the trauma-focused intervention were almost half as likely to report unprotected sex compared to women in the control group [63]. Given the high number of PTEs experienced by young people in South Africa, it is imperative to scale-up such trauma-informed mental health services for adolescents [21].

Community-level interventions addressing harmful gender norms, such as Stepping Stones, have been successful at reducing the perpetuation of intimate partner violence, a significant step forward in reducing HIV transmission and experiences of trauma for adolescent women [48]. For both adolescent men and women, interventions aimed at addressing food insecurities may help to mediate the compounding affects of PTEs on HIV transmission within vulnerable urban environments such as Soweto. This relationship merits further examination. Future interventions should consider the importance of resilience and post-traumatic growth within settings where experiences of traumatic events and HIV risk are extremely high [64].

### Strengths & limitations

In conducting a gender-stratified analysis of PTE occurrence, we demonstrated the multitude of implications that PTEs have on both SRH programs and HIV intervention — informing a gendered approach to addressing PTE and HIV risk. However, we did not include measurements within our survey to assess PTSD symptomology which is a known outcome of experiencing trauma [8, 10, 21], thus we acknowledge this is a limitation of our study which should be further examined within future South African adolescent health studies. Further, we are unable to assess causation within this cross-sectional study. Additional limitations include recall and social desirability bias due to self-reported measures of sexual behaviour and other sensitive topics. In addition, we used a modified variation of the TESI-C; therefore, caution should be used in comparing these findings with other studies using the original version of the TESI-C and other scales similarly measuring experiences of trauma.

## Conclusion

Being an adolescent in Soweto, South Africa poses many challenges: we found a high prevalence of PTEs along with associations highlighting risk for HIV acquisition, particularly for adolescent women. Adolescence is a dynamic and transitional time of the lifecourse, marked by rapid and multiple developmental changes that, through biology and socialization, are distinctly gendered [5, 65, 66]. Enabling and fostering the pathway towards health provides adolescent men and women with a set of meaningful skills and coping mechanisms that they can carry into adulthood [5, 21]. Focusing on preventing multiple co-occurring risks and promoting increased access to mental health services for adolescent men and women facing high exposures to PTEs can begin to address the syndemic of HIV and trauma which pose significant threats to HIV-acquisition, population health and development for South Africa [10].

## Additional file

Additional file 1: Comparison of potentially traumatic event items assessed within the Botsha Bophelo Adolescent Health Survey and TESI-C items. (DOCX 101 kb)

## Abbreviations

AIC: Akaike information criterion
AIDS: Acquired immune deficiency syndrome
aOR: Adjusted odds ratio
ART: Antiretroviral therapy
BBAHS: Botsha Bophelo adolescent health survey
CES-D: Centre for epidemiologic studies depression
HIV: Human immunodeficiency virus
KMAC: Kganya Motsha Adolescent Centre
PHRU: Perinatal HIV research unit
PLHIV: People living with HIV/AIDS
PrEP: Pre-exposure prophylaxis
PTEs: Potentially traumatic events
PTSD: Post-traumatic stress disorder
STIs: Sexually transmitted infections
TasP: Treatment as prevention
TESI-C: Traumatic event screening inventory-child (TESI-C)
TF-CBT: Trauma-focused cognitive behavioural therapy
