# Supplementary material for: Prevalence, type, and correlates of trauma exposure among adolescent men and women in Soweto, South Africa: implications for HIV prevention
Source: BMC Public Health. 2016 Nov 25;16:1191. doi: 10.1186/s12889-016-3832-0 (PMC5123224; doi:10.1186/s12889-016-3832-0)
Supplement: Additional file 1: — Comparison of potentially traumatic event items assessed within the Botsha Bophelo Adolescent Health Survey and TESI-C items. (DOCX 101 kb) [file 12889_2016_3832_MOESM1_ESM.docx]

Supplementary Material.

Comparison of potentially traumatic event items assessed within the Botsha Bophelo Adolescent Health Survey and TESI-C items

|  | Botsha Bophelo Adolescent Health Survey | Traumatic Events Screening Inventory - Child |
| --- | --- | --- |
|  | Have you ever experienced any of the following? |  |
| 1. | Been separated from your mother or the other person who looks after you for more than three months at a time (for example, lived with another relative or in foster care)? | Have you ever been separated from a person who you depend on for love or security for more than a few days OR under stressful circumstances? For example due to foster care, immigration, war, major illness or hospitalization |
| 2. | Have your parents split up or separate? |  |
| 3. | Parents argued frequently or more than usual? |  |
| 4. | Changed schools (not because of graduation) or moved to a new home? |  |
| 5. | Have your parent or guardian lost their job? |  |
| 6. | Lost your home or had no home? |  |
| 7. | Has a family member or someone close to you who had HIV or AIDS? |  |
| 8. | Has a family member or someone close to you who died of HIV or AIDS? |  |
| 9. | Has a family member or someone close to you who died? | Have you ever experienced the death of someone close to him/her |
| 10. | Found out that a family member or someone close to you was very sick or had a bad injury? | Have you ever experienced the severe illness or injury of someone close to you? |
| 11. | Experienced discrimination based on your race or ethnicity? |  |
| 12. | Your family struggled with money that is, struggled to make ends meet? |  |
| 13. | This question is about seeing violence in the streets, in your neighborhood, or at school. Have you ever SEEN an act of violence towards some else, NOT BETWEEN MEMBERS OF YOU AND YOUR FAMILY, such as someone else being attacked, killed, shot at etc. | Have you ever seen or heard people outside your family fighting, hitting, pushing or attacking each other? Or seen or heard about violence such as beatings, shootings or mugging that occurred in setting that are important to you such as school, your neighborhood or the neighborhood of someone important to you? |
| 14. | This question in about violence that may have happened to YOU. This could have been while hanging out, while at school, or in the neighborhood. Have you ever EXPERIENCED an act of violence, NOT BY SOMEONE IN YOUR FAMILY, such as being attacked, etc. | Has someone ever physically assaulted you, like hitting, pushing, choking shaking, biting or burning? Or punished your and caused physical injury or bruises. Or attacked you with a gun, knife, or other weapon//Has someone ever directly threatened you with serious physical harm |
| 15. | Sometimes kids see people in their family getting hurt, being beaten, punched, kicked, chocked, or thrown down hard by other family members (sometimes it is part of a fight). Have you ever SEEN this happen TO SOMEONE ELSE IN YOUR FAMILY? | Has someone ever physically assaulted you, like hitting, pushing, choking shaking, biting or burning? Or punished your and caused physical injury or bruises. Or attacked you with a gun, knife, or other weapon//Has someone ever directly threatened you with serious physical harm |
| 16. | Sometimes kids are hurt by people in their own family, such as being punched, kicked, chocked, or thrown down hard. Have you ever experienced being hurt by someone in your family? | Have you ever seen, heard or heard about people in your family physically fighting, hitting, stabbing, kicking or pushing each other. Or shooting with a gun or stabbing or using any other kind of dangerous weapon//Have you ever seen or heard people in your family threaten to seriously harm each other? |
| 17. | Have you ever deliberately inflicted harm on another person? Check all that apply (Yes/No/Unsure/Prefer not to answer) |  |
| 18. | Has someone ever forced you to have sexual intercourse with them? |  |
| 19. | Have you ever forced someone to have sexual intercourse with you? |  |
|  |  | Have you ever been in a serious accident where someone could have been (or actually was) severely injured or died? |
|  |  | Have you ever seen a serious accident where someone could have been (or actually was) severely injured or died? |
|  |  | Have you ever been in a natural disaster where someone could have been (or actually was) severely injured or died or where |
|  |  | Have you ever undergone any serious medical procedures or had a life threatening illness? Or been treated by a paramedic |
|  |  | Has anyone ever kidnapped you? (Including a parent or relative) Or has anyone ever kidnapped someone close to you? |
|  |  | Have you ever been attacked by a dog or other animal? |
|  |  | Have you ever known or seen that a family member was arrested, jailed, imprisoned or taken away |
|  |  | Have you ever been directly exposed to war, armed conflict, or terrorism? |
|  |  | Have you ever seen or heard acts of war or terrorism on the television or radio? |
